# Smc5/6 Is a Telomere-Associated Complex that Regulates Sir4 Binding and TPE

**DOI:** 10.1371/journal.pgen.1006268

**Published:** 2016-08-26

**Authors:** Sarah Moradi-Fard, Jessica Sarthi, Mireille Tittel-Elmer, Maxime Lalonde, Emilio Cusanelli, Pascal Chartrand, Jennifer A. Cobb

**Affiliations:** 1 Departments of Biochemistry & Molecular Biology and Oncology, Robson DNA Science Centre, Arnie Charbonneau Cancer Institute, Cumming School of Medicine, University of Calgary, Calgary, Alberta, Canada; 2 Département de Biochimie, Université de Montréal, Montréal, Quebec, Canada; St Jude Children's Research Hospital, UNITED STATES

## Abstract

SMC proteins constitute the core members of the Smc5/6, cohesin and condensin complexes. We demonstrate that Smc5/6 is present at telomeres throughout the cell cycle and its association with chromosome ends is dependent on Nse3, a subcomponent of the complex. Cells harboring a temperature sensitive mutant, *nse3*-1, are defective in Smc5/6 localization to telomeres and have slightly shorter telomeres. Nse3 interacts physically and genetically with two Rap1-binding factors, Rif2 and Sir4. Reduction in telomere-associated Smc5/6 leads to defects in telomere clustering, dispersion of the silencing factor, Sir4, and a loss in transcriptional repression for sub-telomeric genes and non-coding telomeric repeat-containing RNA (TERRA). *SIR4* recovery at telomeres is reduced in cells lacking Smc5/6 functionality and vice versa. However, *nse3*-1/ *sir4* Δ double mutants show additive defects for telomere shortening and TPE indicating the contribution of Smc5/6 to telomere homeostasis is only in partial overlap with SIR factor silencing. These findings support a role for Smc5/6 in telomere maintenance that is separate from its canonical role(s) in HR-mediated events during replication and telomere elongation.

## Author Summary

Structural Maintenance of Chromosome (SMC) complexes, include cohesin, condensin, and the Smc5/6 complex. These complexes are involved in many aspects of chromosome organization, with cohesin and condensin having relatively well-characterized roles. Cohesin holds newly replicated DNA strands together and condesin is critical for chromosome condensation and genome compaction as cells enter mitosis. However, a role for the Smc5/6 complex in higher-level chromosome organization has remained ill defined. The Smc5/6 complex is recovered at chromosome ends in all stages of the cell cycle and has a role in telomere biology. Smc5/6 integrity is necessary for Sir4 binding, telomere clustering, and transcriptional silencing. In all, our data suggest that Smc5/6 has a physiologically relevant role in chromatin maintenance at telomeres and telomere organization within the nucleus that are distinct of it functionality in homologous recombination.

## Introduction

Structural maintenance of chromosome (SMC) protein complexes facilitate chromosome structure and organization in eukaryotes. Three SMC complexes are found in eukaryotes and each has a unique role in chromosome dynamics and metabolism. Underscoring their importance and distinct functionality, all three complexes and their individual components are essential for cell viability. Cohesin regulates sister chromatid cohesion and condensin is important for chromosome compaction by tethering different regions of the same chromosome [1–3].

**Fig 1:**
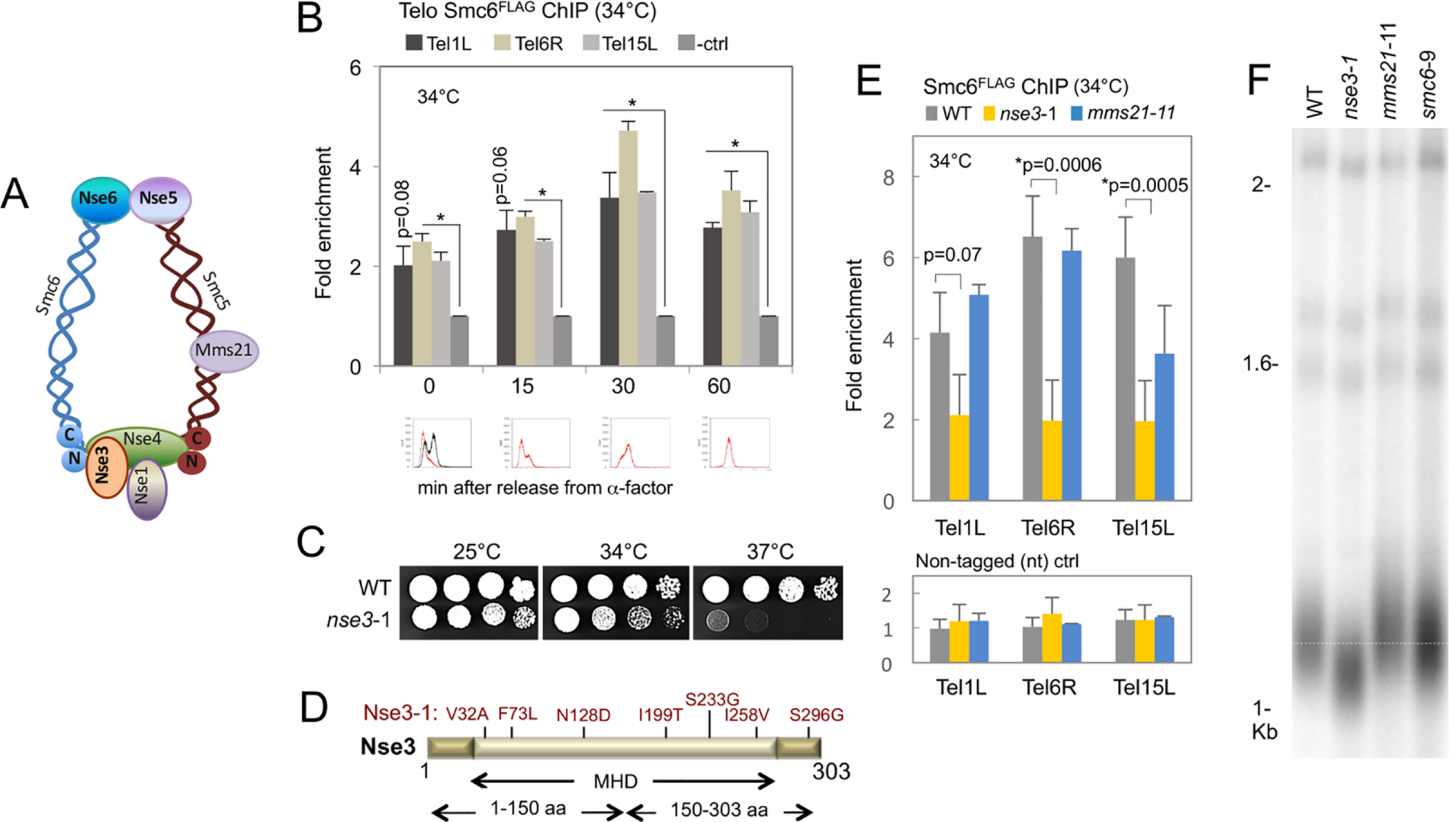
Smc5/6 is a telomere binding complex. (A) A schematic representation of the Smc5/6 complex showing the location of Nse3 as part of a trimeric sub-complex located at the head region where Smc5 and Smc6 meet. (B) Chromatin immunoprecipitation (ChIP) followed by qPCR was performed on Smc6^FLAG^ (JC1594) at the indicated time points after release from α-factor. The fold enrichment at three native subtelomeres (Tel1L, Tel6R and Tel15L) compared to a control (ctrl) late replicating region on Chromosome V (469104–469177) is reported with the mean ± SD for n≥3 experiments performed in technical duplicate. (*) Indicates a statistically significant level of enrichment compared to the ctrl with p values < .05 by a two-tailed *t*-test. Smc6^FLAG^ enrichment at Tel1L is higher at 0 and 15 minutes after release, but with p values = 0.08 and p = 0.06 respectively. The lower panels show flow cytometry on ChIP samples with an asynchronous culture shown in black at the 0 time point. (C) Drop assay of exponentially growing wild type (JC470) and *nse3*-1 (JC3607) cells that were grown for 48 hours at the indicated temperatures on YPAD and 1:5 serial dilutions. (D) Schematic diagram of Nse3. “MHD” represents Melanoma Homology Domain in Nse3 protein. Seven amino acid substitutions in Nse3-1 are shown in red. (E) Chromatin immunoprecipitation (ChIP) on Smc6^FLAG^ in wild type (JC1594), *nse3*-1 (JC2630), *mms21*-11 (JC2075) and the non-tagged (nt) control strains for wild type (JC470), *nse3*-1 (JC3607), and *mms21*-11 (JC1879) in asynchronous cultures. The fold enrichment levels are relative to the late-replicating control region on Chr V for n = 3 experiments with the mean ± SD. All primers are listed in S2 Table. Enrichment levels for wild type and mutant cells with p values < .05 from a two-tailed *t*-test are indicated by (*). (F) Telomere length was determined as previously described [15]. Southern blot analysis was performed on 1μg XhoI-digested genomic DNA hybridized with a radiolabeled poly (GT/CA) probe in wild type (JC471), *nse3*-1 (JC3032), *mms21*-11 (JC1981), and *smc6*-9 (JC1358).In higher eukaryotes, telomeres are challenged by the continuous loss of DNA due to the end replication problem. However, in *Saccharomyces cerevisiae*, telomere length is maintained by the continued expression of telomerase, an enzyme containing a RNA subunit that serves as a template for *de novo* telomere synthesis [16]. After the 3’ end is extended by telomerase, the replicative DNA polymerase fills in the complementary strand. Both telomerase extension and semiconservative replication at telomeres are included in the final events of S phase (for review see [17]). In the absence of telomerase activity, telomeres shorten extensively, leading to senescence, however a small percentage of cells survive by extending their telomeres through the HR dependent alternative lengthening of telomeres (ALT) pathway [18–21].

The third complex, Smc5/6, contains six non-SMC proteins in addition to Smc5 and 6 including Mms21/ non-Smc element 2 (Nse2), which is an E3 SUMO ligase (Fig 1A) [4–6]. As well, Nse1 and Nse3 bind to Nse4 to form a heterotrimer, which in turn interacts with the ATPase head domain generated by the N- and C-termini of Smc5 and Smc6 [7, 8]. Nse1 is a putative ubiquitin ligase and Nse3 is a MAGE (melanoma-associated antigen gene) domain containing protein that is important for loading the complex onto chromatin [9–11]. The Smc5/6 complex functions in homologous recombination (HR) and replication, and it localizes to repetitive elements such as the rDNA and telomeres presumably to promote and resolve HR-dependent intermediates [12–14].

A telomeric function for the Smc5/6 complex in ALT has been demonstrated in both human and yeast cells [22–24]. In human ALT cells, a knockdown of components in the Smc5/6 complex inhibits recombination at telomeres, resulting in telomere shortening and senescence [22]. As well, in telomerase negative yeast cells *smc6*-9 and *mms21*-11 mutant alleles exhibited accelerated senescence attributed to the accumulation of recombination intermediates, but also to an HR–independent mechanism involving the untimely termination of DNA replication [23, 24]. The Smc5/6 complex is enriched at telomeres in telomerase positive asynchronous cultures [12, 13], however its characterization outside the ALT pathway remains limited. In telomerase positive cells, the *smc6*-9 allele exhibited mis-segregation of repetitive elements at telomeres which is attributed to defects in HR [12] and the *mms21*-11 allele was shown to have defects in telomere clustering with increased telomere position effect (TPE) [4]. Subsequent to the initial characterization of *mms21*-11, *mms21*Δsl mutants showed a loss of TPE and SIR binding [25]. Thus, allele specific variations have complicated the understanding of Mms21 and SUMO mediated events in TPE [4, 25]. Further characterization of Smc5/6 in telomere homeostasis using a mutant allele of a distinct complex component will provide additional information about the functionality of Smc5/6 at telomeres.

Telomeric DNA in *S*. *cerevisiae* contains tandem repeats of (AC_1-3_/TG_1-3_) _n_; n = 275–375 [26] along with two types of subtelomeric repeat elements called Y’ and X [27]. The Y’ sequence is located adjacent to the tandem repeats at many, but not all subtelomeres, whereas X-elements are found at the ends of all chromosomes [28]. Rap1 binds directly to the double-stranded TG_1-3_ DNA moiety and is a central regulator of telomere biology [29]. The C-terminal domain of Rap1 interacts with Rif1 and Rif2 and regulates telomere length via a counting system that involves their interaction with Rap1 [30, 31]. Telomeres are elongated in *rif1*Δ and *rif2*Δ cells via telomerase dependent and HR independent events [32, 33].

The C-terminal domain of Rap1 also binds the SIR complex, which is important for transcriptional silencing primarily via interactions with Sir4 [30, 32, 34, 35]. SIR proteins are important for telomere position effect (TPE) and the formation of heterochromatin, which nucleates at telomeres and then spreads several kilobases into subtelomeric regions [36, 37]. Subtelomeric heterochromatin is maintained by seemingly distinct events that are likely to be interrelated *in vivo*. For example, in budding yeast, 32 telomeres cluster together in 3–8 foci at the nuclear periphery, and this drives the sequestration of SIR complex sub-compartments within the nucleus, and promotes silencing [38]. Additionally, the SIR complex, along with Rif1 and Rif2, modulates the level of long non-coding telomeric repeat-containing RNA, TERRA, which is also an integral factor in heterochromatin formation [39–42]. TERRA levels have never been reportedly assessed in Smc5/6 compromised cells and a role for the complex in heterochromatin maintenance and transcription at telomeres remains to be clearly defined.

Here we show that the Smc5/6 complex binds telomeres, not only during late S phase when telomeres are synthesized, but also throughout the cell cycle in telomerase positive cells. Telomere clustering and full Sir4 binding is indeed dependent on the SUMO ligase activity of Mms21, however in the course of characterizing a temperature sensitive (*ts*) mutant of NSE3, telomere defects were observed in cells harboring the *nse3*-1 allele, which have not been previously reported with other alleles having compromised Smc5/6 functionality. TPE and TERRA regulation, as well as telomere length defects in *nse3*-1 mutants were additive with the loss of *SIR4*. In all, our data support a model that extends the functionality of Smc5/6 at telomeres beyond its previously reported roles in homology-mediated events in the ALT pathway [22–24].

## Results

### The Smc5/6 complex is constitutively bound to telomeres and reduced in *nse3*-1 mutant cells

The Smc5/6 complex has been detected at telomeres [12, 13] and stalled and collapsed replication forks [43–48]. Given that telomeres are difficult to replicate sites and prone to fork stalling, we wanted to determine if the presence of Smc5/6 at chromosome ends coincided solely with telomere duplication or if it was present at telomeres independent of replication. We monitored Smc6^FLAG^ enrichment as a marker for the complex and performed chromatin immuno-precipitation (ChIP)–qPCR at multiple time points after cells were synchronously released from G1 into S phase. Significant enrichment of Smc6^FLAG^ was observed at three telomeres above a late-replicating control region on Chr V (Fig 1B) [49, 50], showing the Smc5/6 complex is constitutively present at telomeres and not only during the time of telomere replication in late S phase (Fig 1B).

It was recently demonstrated that Nse3 in fission yeast is important for loading the Smc5/6 complex onto chromatin [11]. We wanted to determine the involvement of Nse3 in localizing Smc5/6 to its endogenous binding sites such as telomeres in budding yeast. As with all subcomponents of the complex (Fig 1A), *NSE3* is essential precluding its deletion. Therefore, we utilized a mutant allele, *nse3*-1, which contains seven amino acid substitutions and was isolated from a screen for temperature sensitivity (*ts*) at 37°C [51] (Fig 1C and 1D).

As *nse3*-1 mutant cells do not synchronize efficiently with α-factor (S1A Fig), we determined Smc5/6 localization in asynchronous cultures at the semi-permissive temperature 34°C. The enrichment of Smc6^FLAG^ was significantly reduced in *nse3*-1 mutant cells at telomeres and other known sites of Smc5/6 binding (Fig 1E, S1B Fig). In contrast, the level of Smc6^FLAG^ recovered at telomeres in *mms21*-11 mutant cells, which are HR and SUMO ligase deficient, was similar to wild type (Fig 1E, S1B Fig). One explanation for the loss of Smc6^FLAG^ recovery is that the complex is unstable in *nse3*-1 mutant cells. To address this possibility, we performed co-immunoprecipitation with two subcomponents that do not directly interact with one another, Nse6 and Smc5, as previously described [48]. In *nse3*-1 mutant cells, Nse6 was recovered in Smc5 pull-downs at levels comparable to wild type cells (S1C Fig), suggesting the complex does not markedly dissociate in *nse3*-1 mutants.

Telomeres were also slightly shorter in *nse3*-1 mutants compared to wild type and HR-defective *smc6*-9 mutant cells (Fig 1F). In contrast, slightly longer telomeres were observed in *mms21*-11 mutants (Fig 1F), which is consistent with its initial characterization showing that this allele had longer telomeres [4]. The changes are indeed subtle, however there is a noticeable difference in telomere length when comparing the *nse3*-1 to the other complex mutants, suggesting that the Smc5/6 complex might have a role at telomeres distinct from HR-mediated events.

### The Smc5/6 complex is important for telomere clustering

**Fig 2:**
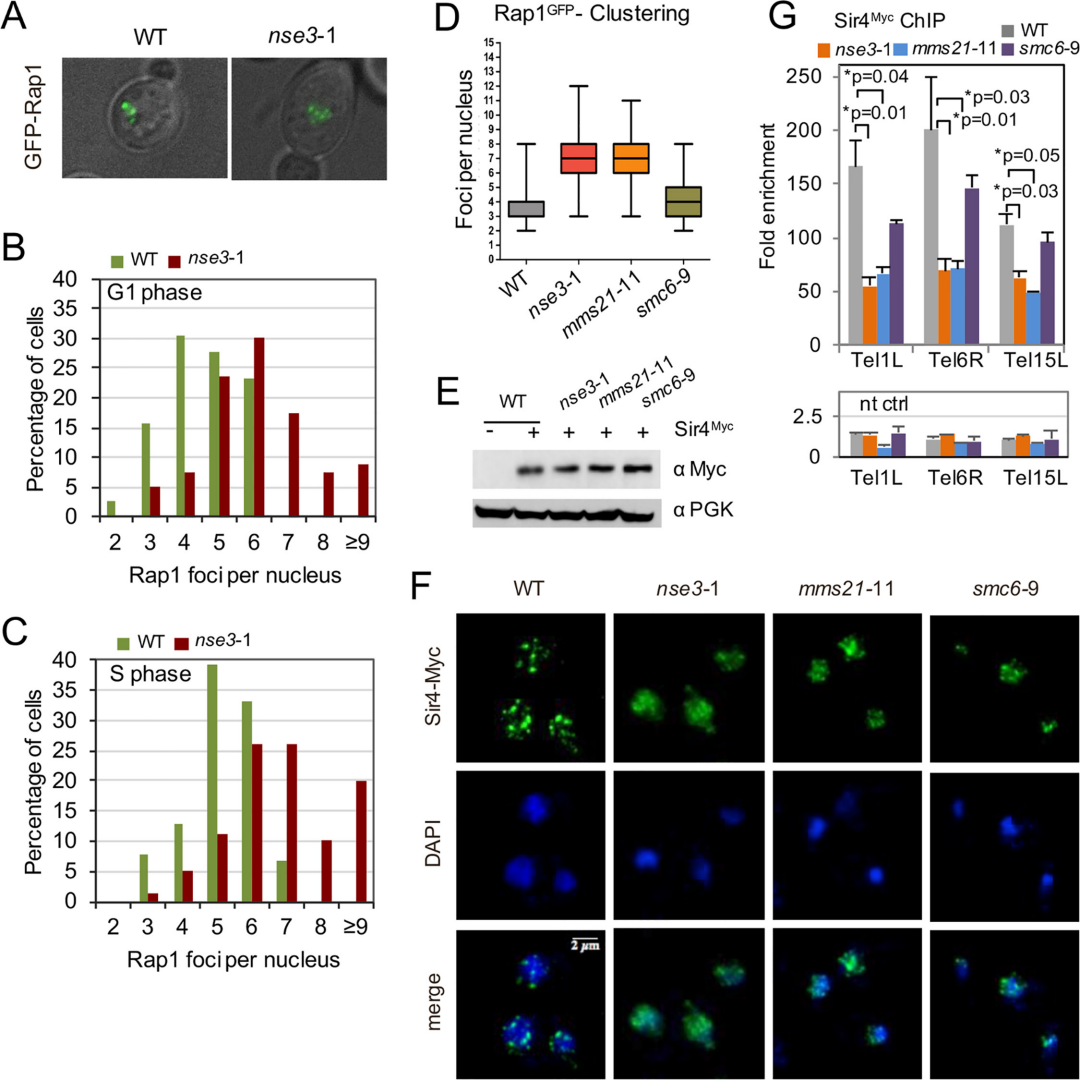
Smc5/6 is critical for telomere clustering and Sir4 binding to telomeres. (A) Rap1-GFP foci in WT (JC1822) and *nse3*-1 (JC3041) cells counted as a measurement for telomere clustering with representative merged images of GFP and DIC channels. (B-C) The number of GFP-Rap1 foci was determined for cells within G1 (unbudded) or S (small budded cells) phases in at least 100 cells for each cell cycle stage, and (D) compared with *mms21*-11 (JC1827) and *smc6*-9 (JC2710). (E-F) Western blot analysis and immunofluorescence staining using α-Myc antibody (green in IF) to detect Sir4^Myc^ in WT (JC3433), *nse3*-1 (JC3452), mms21-11 (JC3597), and smc6-9 (JC2907) cells with DAPI staining shown in blue. (G) ChIP was performed on Sir4^Myc^ as in Fig 1E from asynchronous cultures and in more than one isogenic strain if available. The fold enrichment for each strain is calculated for n≥3 experiments with the mean ± SD at three native subtelomeres (Tel1L, Tel6R and Tel15L). The p values < 0.05 from a two-tailed *t*-test are indicated by (*) for wild type (JC2671 and JC3433), *nse3*-1 (JC3452 and JC3849), *mms21*-11 (JC3597), and *smc6*-9 (JC2907 and JC3087) and non-tagged (nt) control strains included wild type (JC470), *nse3*-1 (JC3607), *mms21*-11 (JC1879), and *smc6*-9 (JC1358).

Telomere clustering at the nuclear periphery in *S*. *cerevisiae* establishes sub-nuclear zones that sequester repressors of transcription [52, 53]. Clustering can be visualised by performing immunofluorescences and counting GFP-Rap1 foci. In haploid cells, it has been demonstrated that 32 telomeres cluster in limited number [54], and consistent with this, our quantification showed ~90% of wild type cells contained ≤ 6 foci in both G1 and S phases of the cell cycle at 34°C (Fig 2A–2C). In contrast, *nse3*-1 mutants had ≥ 6 foci in ~65% and ~80% of the cells in G1 and S phases respectively, with 10–20% having ≥ 9 foci (Fig 2A–2C). In a side-by-side comparison and in line with its initial characterization, a similar clustering defect was observed in *mms21*-11 mutants [4], but *smc6*-9 mutant cells were similar to wild type (Fig 2D).

Defects in clustering coincide with a disruption in SIR proteins, [55, 56]. Sir4^Myc^ is expressed at similar levels in all strains (Fig 2E), and as measured by immunofluorescence, Sir4^Myc^ forms discrete punctate foci in wild type cells (Fig 2F). In contrast, Sir4^Myc^ became relatively dispersed throughout the nucleus in *nse3*-1 mutant cells (Fig 2F). Dispersion was also observed in *mms21*-11 and *smc6*-9 alleles, but to a lesser extent than the level observed in *nse3*-1 mutants (Fig 2F). Foci, albeit with reduced intensity, remained in all mutants to varying degrees, therefore as a complement to immunofluorescence and to quantify changes at telomere, we performed ChIP with Sir4^Myc^. The level of Sir4^Myc^ recovered at telomeres in both *nse3*-1 and *mms21*-11 mutants was reduced to ~40% that of wild type cells (Fig 2G). In *smc6*-9 mutant cells, the level of Sir4^Myc^ at telomeres was not significantly different from the amount recovered in wild type (Fig 2G). Taken together, the alleles with defects in clustering, *nse3*-1 and *mms21*-11, also showed a reduction in the level of Sir4 bound at telomeres.

Sir4 sumoylation by Siz2 was previously implicated in peripheral telomere position [57, 58]. Given that our results indicated Sir4 localization to be regulated by Mms21, we investigated if the SUMO status of Sir4 itself might provide a level of regulation. Similar to *siz2*Δ, the level of Sir4 sumoylation was reduced in *mms21*-11, however SUMO levels remained similar to WT, if not higher in *nse3*-1 mutants (S2 Fig). These data suggest that Sir4 localization to telomeres is not regulated by the SUMO status of Sir4 in *nse3*-1 cells.

### The Smc5/6 complex binding to telomeres is regulated by Sir4 and is important for TPE

**Fig 3:**
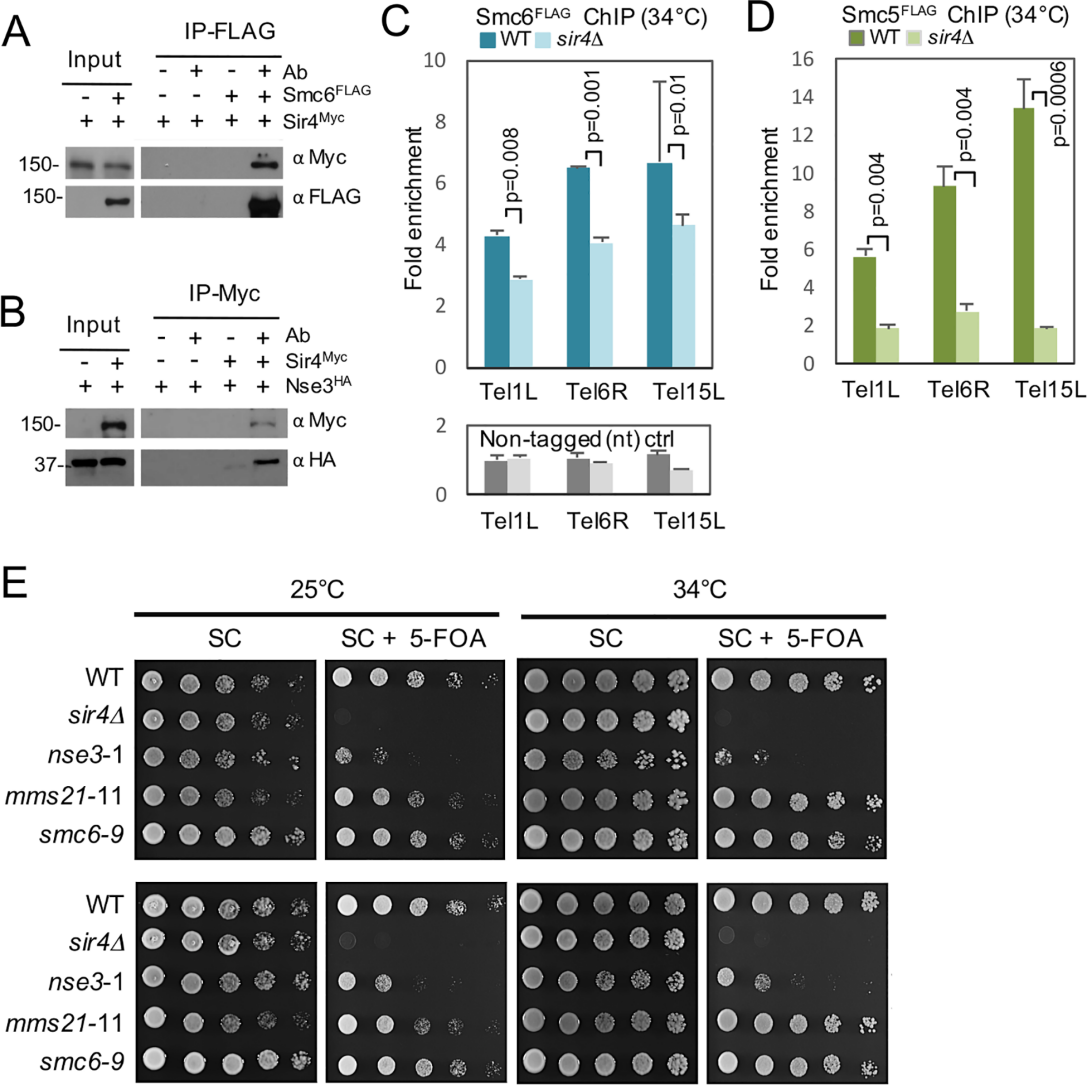
Smc5/6 physically associate with Sir4 and is important for TPE. (A) Co-immunoprecipitation (Co-IP) as described in the materials and methods section was performed in cells carrying Sir4^Myc^ and Nse3^HA^ (JC3736) with Nse3^HA^ (JC2823) as control or (B) Sir4^Myc^ and Smc6^FLAG^ (JC3853) with Smc6^FLAG^ (JC1594) as a control. (C) ChIP was performed on Smc5^FLAG^ in wild type (JC3728) and *sir4*Δ (JC3720) and (D) Smc6^FLAG^ in wild type (JC1594) and *sir4*Δ (JC3732) and non-tagged (nt) strains in wild type (JC470) and *sir4* Δ (JC3737) as described in Fig 1E. The fold enrichment levels are relative to the late-replicating control region on Chr V for n≥3 experiments with the mean ± SD at three native subtelomeres (Tel1L, Tel6R and Tel15L) with p values < .05 from a two-tailed *t*-test indicated. (E) TPE was determined in strains with the *URA3* reporter at the *adh4* locus of Chromosome VIIL. Tenfold (1:10) serial dilutions of overnight cultures were spotted onto SC (complete medium) and SC + .1% 5-FOA plates at 25°C and 34°C in wild type (JC1991), *sir4*Δ (JC3818), *nse3*-1 (JC3860), *mms21*-11 (JC1080) and *smc6*-9 (JC1077) isogenic strains.

To further understand the relationship between Sir4 and the Smc5/6 complex we performed co-immunoprecipitation to see if we could detect a physical interaction. Upon Smc6^FLAG^ immunoprecipitation, we recovered Sir4^Myc^ (Fig 3A). We had variable results with the reciprocal IP, however we found that Nse3^HA^ associated with Sir4^Myc^ pull downs (Fig 3B), suggesting that the Smc5/6 and SIR complexes physically associate *in vivo*.

The Smc5/6 complex influenced Sir4 recovery at telomeres and a physical interaction between the complexes was detected. Thus, the reverse was performed to determine if Sir4 levels impacted the localization of Smc5/6 at telomeres. ChIP was performed with Smc6^FLAG^ and Smc5^FLAG^ and recovery at telomeres was compared in *sir4*Δ and wild type cells (Fig 3C and 3D). The level of Smc6^FLAG^ in cells lacking *SIR4* decreased to ~60% the amount recovered in wild type cells (Fig 3C). Similarly, Smc5^FLAG^ was reduced in *sir4*Δ mutants to ~25% that of wild type levels (Fig 3D). As Smc5 and Smc6 are present at stoichiometric levels in the complex [4, 59], the greater relative change with Smc5^FLAG^ might result from IP variability. Nonetheless, there is a statistically significant decrease in both core factors of the Smc5/6 complex bound to telomeres in *sir4*Δ mutants compared to wild type cells (Fig 3C and 3D).

**Fig 4:**
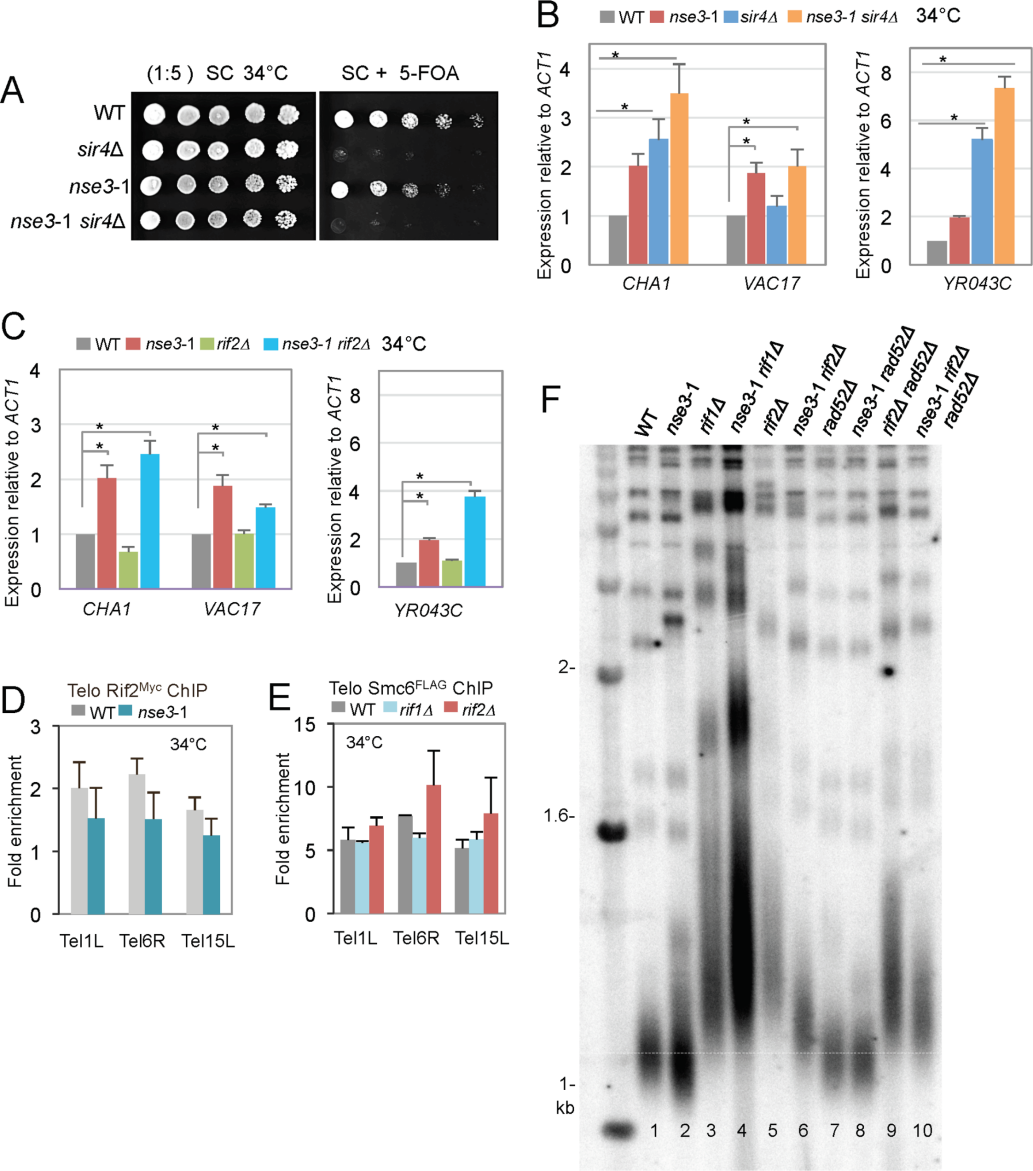
The *nse3*-1 allele exhibits genetic interactions with the loss of *SIR4* and *RIF2*. (A) TPE was determined in strains with the *URA3* reporter at the *adh4* locus of Chromosome VIIL as in Fig 3E. Overnight cultures were spotted onto SC (complete medium) and SC + .1% 5-FOA plates at 34°C in wild type (JC1991), *sir4*Δ (JC3818), *nse3*-1(JC3860), *nse3*-1 *sir4*Δ (JC3870) isogenic strains. (B) Transcription levels in wild type (JC470), *nse3*-1 (JC3607), *sir4*Δ (JC3737), and *nse3*-1 *sir4*Δ (JC3741), and (C) *rif2*Δ (JC2992) and *nse3*-1 *rif2*Δ (JC3269) at sub-telomeric genes *CHA1* and *VAC17* on Tel3L and *YR043C* on Tel9R as described in [62, 63]. Expression values are mRNA levels relative to *ACT1* and normalization to wild type cells. Error bars represent ± SD of n = 3 experiments with p values < .05 from a two-tailed *t*-test indicated by (*). (D) Chromatin immunoprecipitation (ChIP) was performed on Rif2^Myc^ and showed similar levels of recovery in wild type (JC2380) and *nse3*-1 (JC3235) mutants. (E) ChIP on Smc6^FLAG^ in wild type (JC1594), *rif1*Δ (JC2754) and *rif2*Δ (JC3074) cells with enrichment levels for untagged strains in wild type and mutants shown in S5C and S5D Fig. The mean ± SD of the fold enrichment at three native subtelomeres (Tel1L, Tel6R and Tel15L) relative to the control (ctrl) late replicating region on Chromosome V (469104–469177) is reported. In *rif2Δ* mutants the p values < .05 = 0.53 (Tel1L), 0.13 (Tel6R), and 0.15 (Tel15L) indicated that the difference was not significant from wild type. (F) Telomere length was determined as previously described [15]. Southern blot analysis was performed on 1μg XhoI-digested genomic DNA hybridized with a radiolabeled poly (GT/CA) probe in wild type (JC470), *nse3*-1 (JC3607), *rif1*Δ (JC3448), *nse3*-1 *rif1*Δ (JC3623), *rif2*Δ (JC2992), *nse3*-1 *rif2*Δ (JC3269), *rad52*Δ (JC1427), *nse3*-1 *rad52*Δ (JC3629), *rif2*Δ *rad52*Δ (JC3603), and *nse3*-1 *rad52*Δ *rif2*Δ (JC3627) strains.

Sir4 is a critical factor for TPE and in the maintenance of heterochromatin near telomeres [60]. As the Smc5/6 complex interacts with Sir4, and the presence of Smc5/6 is important for Sir4 recovery at telomeres and vice versa, we assessed a role for the complex in transcriptional gene silencing regulation. TPE was determined in reporter strains where *URA3* was integrated at the left arm of telomere VII [61]. Consistent with previous reports, *sir4*Δ cells showed defects in TPE as measured by their compromised ability to form colonies on medium containing 5-fluoroorotic acid (5-FOA) (Fig 3E) [60]. For *nse3*-1 mutants, TPE was disrupted but not to the level observed with *sir4*Δ (Fig 3E). In contrast and consistent with previous reports, TPE in *mms21*-11 and *smc6*-9 mutant cells remained intact at 25°C and 34°C (Fig 3E; [4]). This data indicated that the loss of silencing in *nse3*-1 mutant cells could not be solely attributed to a defect in Sir4 recruitment. This is supported by the observation that both *nse*3-1 and *mms21*-11 mutants showed a comparable defect of Sir4 recovery at telomeres and this was sufficient to silence the reporter transgene in the *mms21*-11 allele.

### The Smc5/6 complex contributes to telomere homeostasis and interacts genetically with *SIR4* and *RIF2*

To bring insight to the functionality of Smc5/6 in transcriptional silencing at telomeres the *nse3*-1 allele was combined with the loss of either *SIR4* and/or *RIF2*. Utilizing the *URA3* reporter assay (Fig 4A, S3 Fig), it was difficult to observe an additive defect in silencing for *nse3*-1 *sir4*Δ double mutants because the loss of silencing is so penetrant with the loss of *SIR4*. Therefore, two endogenous sub-telomeric sites, *YR043C* and *CHA1*, on Tel9R and Tel3L respectively were assessed [62, 63]. Gene transcription increased in *nse3*-1 *sir4*Δ double mutants compared to *sir4*Δ single mutant cells (Fig 4B). Moreover, a defect in silencing was also observed in *nse3*-1 mutants at *VAC17*, a gene adjacent to *CHA1* and previously determined to be silenced independently of Sir4 (Fig 4B; [63]). An additive loss of silencing was not observed when *smc6*-9 was combined with *sir4*Δ (S4 Fig), suggesting that HR-regulated functions involving the Smc5/6 complex are separable from its function in transcriptional silencing. In *rif2*Δ cells, silencing remains and even increases presumably through increased binding of Sir4 to Rap1 at telomeres (Fig 4C) [30, 64]. The *nse3*-1 *rif2*Δ double mutants exhibited a loss of silencing that was similar to *nse3*-1 single mutant cells (Fig 4C), however this was difficult to observe when measuring TPE from the *URA3* reporter unless cell concentrations were low (S3 Fig).

Rap1 binds both Sir4 and Rif1/2 [30, 32, 34, 65], and given the interactions *nse3*-1 had with these factors it was important to assess Rap1 binding to telomeres in *nse3*-1 mutants. By ChIP, we observed no significant difference in the level of Rap1^Myc^ bound at telomeres in *nse3*-1 mutants compared to the levels in wild type cells (S5A Fig). These data also support the interpretation that the increased number of Rap1 foci we measured in *nse3*-1 cells resulted from a disruption in telomere clustering rather than a disruption of Rap1 binding to telomeres (Fig 2A–2C).

Nse3 was previously reported to interact with Rif2 in a high-throughput yeast two-hybrid (Y2H) screen [66]. We verified the Rif2-Nse3 interaction and determined it was reduced when *nse3*-1 was expressed (S6 Fig), however, in contrast to Sir4^Myc^, the levels of Rif1^Myc^ and Rif2^Myc^ at telomeres in *nse3*-1 were similar to wild type (Fig 4D, S5B Fig), and no significant change with Smc6^FLAG^ was measured at telomeres in cells lacking *RIF1* or *RIF2* (Fig 4E). In all, these data suggest that the physical association between Nse3 and Rif2 is not driving the recruitment of either factor/complex to telomeres.

Cells carrying the *nse3*-1 allele exhibit slightly shorter telomeres (Figs 1F and 4F), which is opposite to cells lacking *RIF1* or RIF2, which are negative regulators of telomerase [33]. Telomere length was determined when *nse3*-1 was combined with *rif1*Δ and *rif2*Δ. The *nse3*-1 *rif2*Δ double mutant cells exhibited a partial reversion in the telomere length phenotype (lanes 5 and 6; Fig 4F). However, when *nse3*-1 was combined with *rif1*Δ, telomere length looked indistinguishable from *rif1*Δ single mutants (lanes 3 and 4; Fig 4F). These data suggest the *nse3*-1 mutation does not counteract telomere elongation as a general mechanism *per se* and support the model that Rif1 and Rif2 having non-overlapping roles in telomere maintenance even though they interact with each other and with Rap1 [67–70].

As the Smc5/6 complex is implicated in HR and the ALT pathway, we also investigated if the partial reversion of long telomeres in *nse3*-1 *rif2*Δ was regulated by HR events. Upon disruption of *RAD52*, no detectable changes were observed, as telomeres for *nse3*-1 *rad52*Δ and *nse3*-1 *rif2*Δ *rad52*Δ mutants were similar in size to *nse3*-1 and *nse3*-1 *rif2*Δ mutants respectively (lanes 2 and 8; lanes 6 and 10; Fig 4F). Moreover, telomere shortening was not observed when the loss of *RIF2* was combined with the HR-deficient *smc6*-9 allele (S7 Fig). Taken together, these data provide additional support for Smc5/6 having a role at telomeres distinct of its functionality in HR-mediate events.

### TERRA regulation is altered in nse3-1 mutants

In addition to the transcription of gene-coding regions, RNA polymerase II also transcribes TERRA at telomeres [40]. There are reported correlations between non-physiological increases and decreases in TERRA levels with telomeric abnormalities [39, 71]. Moreover, TERRA expression was previously demonstrated to be regulated by Rap1, the SIR complex, and Rif1/2 proteins, with the role of Rif2 being minimal and only at a subset of telomeres [42]. As the *nse3*-1 mutation results in a loss of silencing at subtelomeric genes and showed interactions with Rif2 and Sir4 we measured TERRA expression in cell carrying the *nse3*-1 allele.

**Fig 5:**
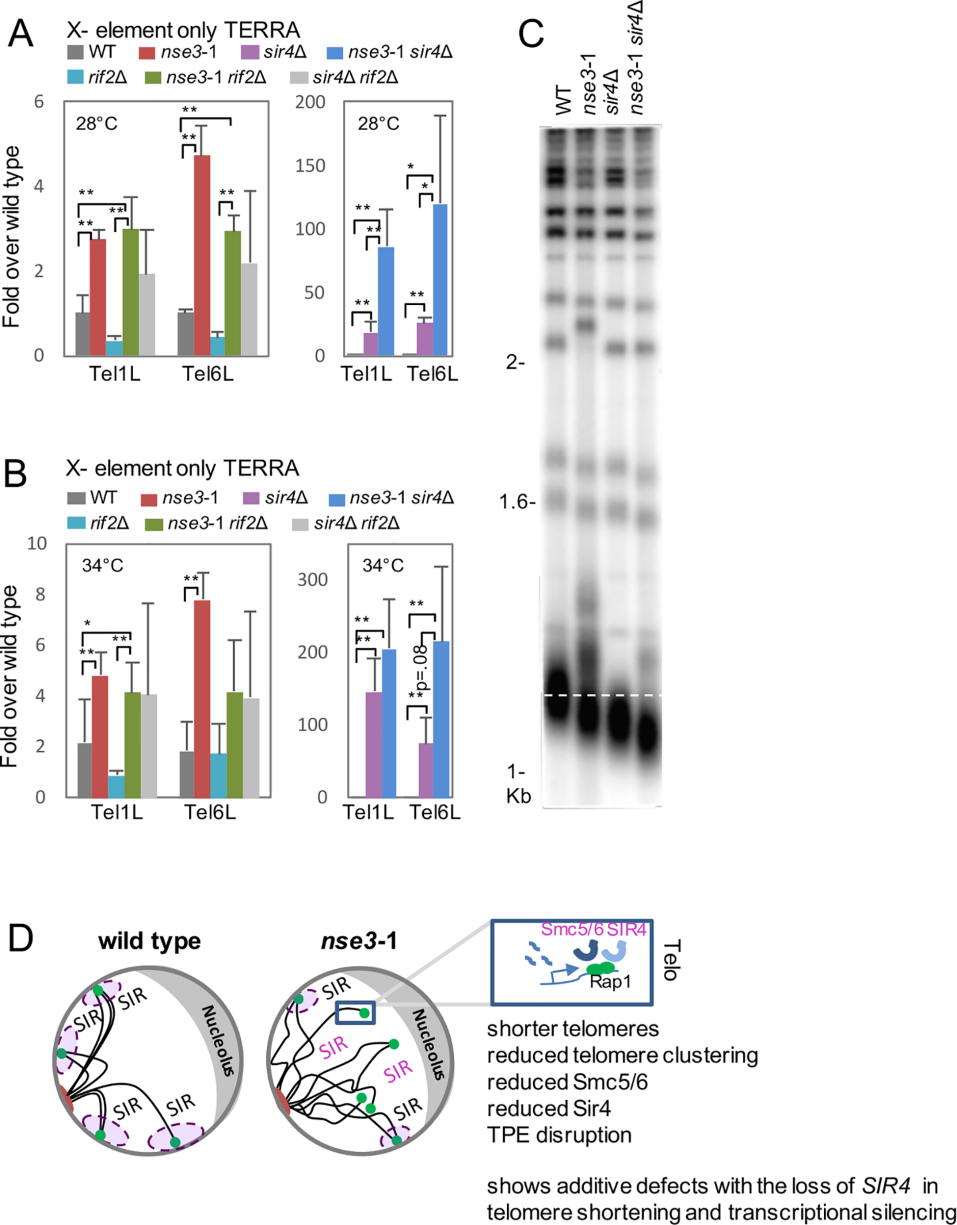
Increases in TERRA and telomere shortening are additive in *nse3*-1 *sir4*Δ double mutant cells. (A and B) TERRA expression was determined by RT-qPCR for Tel1R and Tel6R, X only telomeres, at 28°C and 34°C in wild type (JC470), *nse3*-1 (JC3607), *rif2*Δ (JC2992), *nse3*-1 *rif2*Δ (JC3269), *sir4*Δ (JC3737), *nse3*-1 *sir4*Δ (JC3741), and *sir4*Δ *rif2*Δ (JC3738). TERRA expression from Y’ telomeres is shown in S8 Fig. Statistical significance with p values < .05 (*) or < .01(**) are reported from a two-tailed *t*-test. (C) Telomere length was determined as in Fig 1F by Southern blot analysis on 1μg XhoI-digested genomic DNA hybridized with a radiolabeled poly (GT/CA) probe in wild type (JC470), *nse3*-1 (JC3607), *sir4*Δ (JC3737), and *nse3*-1 *sir4*Δ (JC3741). (D) A model comparing telomere organization in wild type and *nse3*-1 mutants. The Smc5/6 complex localizes to telomeres but significantly decreases in *nse3*-1 mutants (Fig 1E). Moreover, *nse3*-1 alleles exhibit shorter telomeres, reduced telomere clustering, reduced Sir4 binding and defects in TPE. When *nse3*-1 is combined with the loss of *SIR4*, the resulting double mutant cells show additive defects in transcriptional repression and telomere shortening.

Compared to wild type, there was a significant de-repression in TERRA expressed from both X only and Y’ telomeres in *nse3*-1 mutants at both 28°C and 34°C (red, Fig 5A and 5B, S8 Fig). Consistent with previous reports [42], *sir4*Δ mutants showed substantial TERRA expression from X only telomeres (purple, Fig 5A and 5B), and we observed no distinguishable increase in TERRA levels in cells lacking *RIF2* at TEL1R, 6R, or Y’ (aqua, Fig 5A and 5B, S8 Fig). TERRA levels in *nse3*-1 and *nse3*-1 *rif2*Δ were similar and significantly higher than the level measured in *rif2*Δ mutant cells (red, green, and aqua; Figs 5A and 5B and S8). Interestingly, and consistent with the TPE reporter assay, TERRA levels in *sir4*Δ *rif2*Δ cells (light grey) were silenced to levels not statistically different from wild type (dark grey), and similar to *rif2*Δ (aqua, Fig 5A and 5B). There was a 2- and 4- fold increase in the level of TERRA from Y’ and X-only telomeres respectively in *nse3*-1 *sir4*Δ cells (blue) compared to cells lacking *SIR4* (purple) at 28°C (Fig 5A, S8A Fig). The same trend was observed at 34°C, however variability between experiments resulted in p values > 0.05 (Fig 5B, S8B Fig).

Both *nse3*-1 and *sir4*Δ mutants have slightly shorter telomeres (Figs 1F and 5C) [53]. As well, transcription and TERRA levels increased in *nse3*-1 and these phenotypes were additive with *sir4*Δ. Given the correlations between increased TERRA levels and induced transcription with telomere shortening [40, 72] we proceeded to assess telomere length in *nse3*-1 *sir4*Δ double mutants. Telomeres shorten further in double mutants compared to cells harboring either *nse3*-1 or *sir4*Δ single mutant alone (Fig 5C). Highlighting the difference again between *nse3*-1 and *smc6*-9, the level of TERRA expression was not additive in *smc6*-9 *sir4*Δ double mutant cells (S9A and S9B Fig) and in contrast to *nse3*-1, telomere length in *smc6*-9 did not result in additive shortening when combined with *sir4*Δ. (S9C Fig). Taken together, our data support a model whereby Smc5/6 has a role in transcriptional silencing and telomere length maintenance that is different from its involvement in HR dependent events at telomeres and underscore the value of characterizing various *ts* alleles of the complex.

## Discussion

We report a previously uncharacterized function for the Smc5/6 complex with links to transcriptional silencing and demonstrate a role for the complex in telomere homeostasis. In cells carrying the *nse3*-1 allele, Smc5/6 complex levels are markedly reduced at telomeres. This was true for cells grown at 25°C or 34°C, the temperature we used in many of our measurements, indicating that higher temperature did not introduce confounding defects to the complex in this mutant background (S10 Fig). Utilizing *nse3*-1, we show that Smc5/6 is critical for 1. Maintaining proper telomere length, 2. Telomere clustering, 3. SIR complex recovery at telomeres, 4. TPE, and 5. Regulating TERRA levels.

Telomere defects involving mutations in the Smc5/6 complex were first reported with the *mms21*-11 allele; however, the loss of SUMO ligase activity did not appear to impact TPE, as expression from a *URA3* reporter construct integrated at Tel5R remained silent [4]. Upon characterization of the *nse3*-1 allele, we also observed a loss of clustering, but unlike *mms21*-11, TPE was disrupted as shown by an increase in expression of sub-telomeric genes and *URA3* reporter expression. Further characterization of *nse3*-1 and *mms21*-11 alleles demonstrated that a decrease in Sir4 binding at telomeres was common to both alleles (a summary of phenotypes can be found in S3 Table). In agreement with previous reports (Zhao & Blobel, 2005), and in side-by-side comparison with *nse3*-1 and wild type, we find silencing at sub-telomeres remained intact for *mms21*-11 and *smc6*-9 mutants (Fig 3E), suggesting that the partial reduction in Sir4 at telomeres in *mms21*-11 and *nse3*-1 mutants was not sufficient to abrogate silencing. These data also raise the possibility that the complex might have additional functions, which are disrupted in *nse3*-1, that are important for silencing. Our data also suggest a partial interdependency between the Smc5/6 complex and Sir proteins at telomeres. Indeed, a physical interaction is detected between the Smc5/6 complex and Sir4 (Fig 3A and 3B) and in the absence of *SIR4* there is a moderate but statistically significant ~30% reduction in the levels of Smc6^FLAG^ recovered at telomeres, however for comparison, Smc6^FLAG^ was reduced further in *nse3*-1 mutant cells by ~60% the levels of wild type (S10B Fig). Even though Smc5/6 and Sir4 contribute to the stability of one another at telomeres, the defects in TPE and TERRA expression associated with the loss of Smc5/6 at telomeres are additive with the loss of SIR4.

Live-cell imaging at the single-cell level demonstrated that when telomeres become critically short, TERRA is transcribed, and this recruits telomerase to the TERRA-expressing telomere to promote elongation [73]. Increased TERRA levels above physiologically important levels likely have an inhibitory affect on telomere length maintenance. TERRA levels in *nse3*-1 mutants are above wild type and when combined with *sir4*Δ, the double mutants show an even greater increase in TERRA compared to the levels measured in cells lacking *SIR4* only. The elevated transcription and loss of TPE in *nse3*-1 is likely to have a direct effect on TERRA expression and supports the model that Smc5/6 functionality is important for silencing, and when deregulated, transcription lead to increases in TERRA and telomere loss [74]. Telomere shortening is additive in *nse3*-1 *sir4*Δ mutants. The robust expression of TERRA in *nse3*-1 *sir4*Δ cells possibly reinforces the shortening of telomeres, and vice versa. Indeed this explanation is consistent with previous work showing that when TERRA increases, telomeres shorten via telomerase inhibition [40], as well as disrupting the inhibitory effect of yKu70/80 on Exonuclease 1, leading to its increased activity at telomeres [75]. A more speculative model, that will require additional investigation, is that increases in TERRA expression might lead to increased RNA-DNA hybrids at telomeres and subsequently more aberrant replication fork structures that fail to be resolved by Smc5/6, and this results in telomere loss specifically in alleles deficient in silencing, as in *nse3*-1 and *nse3*-1 *sir4*Δ mutant cells. Lastly, an alterative model that we cannot exclude is that there is a more direct effect of *nse3*-1 on telomere length independent of TERRA, which might involve interactions of the Nse1-Nse3-Nse4 sub-complex within Smc5/6 that become altered in cells carrying the *nse3*-1 allele.

We also assessed the SUMO status of Sir4 and determined that sumoylation was reduced in *mms21*-11 mutant cells to levels similar to those previously observed in cells lacking *SIZ2* (S2 Fig) [57]. However, Sir4 sumoylation remained, and was slightly higher in cells harbouring the *nse3*-1 allele when silencing is reduced. This is consistent with previous work showing that increased levels of Siz2, and by extension elevated sumoylation, function antagonistically to silencing [58], and also suggests there is no direct correlation between Sir4 sumoylation in telomere clustering at the periphery. These data are also consistent with the observation that a SUMO-Sir4 fusion construct could not restore anchoring in *siz2Δ* mutants, which suggested that sumoylation of another target, besides Sir4, is important for telomere positioning at the periphery [57].

Telomere clustering and silencing are distinguishable functions [76, 77]. Our data indicates that Smc5/6 likely contributes to both and independently of HR as *smc6*-9 was not distinguishable from wild type in all measures, and that Mms21 sumoylation is important for clustering, but not silencing. Determining the role of Smc5/6 in clustering at the periphery will require further investigation. Organization of telomeres at the periphery is driven by partially redundant pathways involving Sir4 binding to membrane bound Esc1 and Yku70/80 [76, 78]. First, although Sir4 sumoylation does not control clustering we have not assessed if Esc1, which is also a target of sumoylation, regulates clustering in a pathway dependent on Mms21 activity [79, 80]. Secondly, unlike Sir4, we observed that the level of YKu70 at telomeres in *nse3*-1 mutant cells was not statistically different from wild type cells (S11 Fig). However, determining if Mms21 dependent sumoylation of yKu70 at telomeres is critical for Smc5/6 mediated anchoring will provide an additional level of understanding as both Yku70 and Yku80 sumoylation are important for perinuclear positioning [57], and while Yku80 sumoylation is markedly reduced in *siz2Δ* mutants, Yku70-sumoylation is primarily dependent on Mms21 [4, 57]. Our data support a model where the Smc5/6 complex, like other proteins involved in DNA repair, such as Tel1 and Mre11, contributes to transcriptional silencing via two pathways, one involving direct interactions with SIR factors and the other regulating nuclear position and association with the periphery [81].

The current study demonstrates a role for Smc5/6 complex in telomere maintenance that is distinct from its previously characterized functions in replication and HR. Our data show that the Smc5/6 complex is a *bona fide* telomere-binding factor that has reduced recovery in *nse3*-1 mutant cells (Fig 5D). Our study establishes Smc5/6 as having a physiological role in the structural maintenance of chromosome ends where its localization and integrity contribute to the stabilization of factors with well-established roles in telomere maintenance and metabolism. Consistent with a role in end protection, the localization of Smc5/6 to telomeres is critical for telomere clustering and transcriptional repression (Fig 5D). These roles for Smc5/6 together its involvement in the various aspects of HR-mediated DNA metabolism, such as replication and repair, perhaps contribute to the essential requirement of this complex for cell survival.

## Materials and Methods

### Yeast strains and plasmids

All strains used in this study are listed in S1 Table. The *nse3*-1 mutant was a kind gift from Dr. P. Hieter at Michael Smith Laboratories. In all experiments exponentially growing cells were incubated at 34°C for 2hrs before harvesting, unless indicated otherwise. Drop assays were performed by growing cells overnight, and then performing 10-fold serial dilutions where 4μl of each dilution were plated on YPAD an incubated at the indicated temperature. For repression assays, 5-fold or 10-fold dilutions from overnight cultures were plated on SC or SC + 5-FOA as described [76, 82] at the indicated temperatures.

### Chromatin immunoprecipitation (ChIP)

ChIP experiments performed as described previously [83], except that cells were incubated at 34°C for 2 hours before crosslinking with formaldehyde in media where the temperature was held a 25°C to allow efficient crosslinking. Immunoprecipitates were washed once with lysis buffer (50 mm HEPES, 140 mm NaCl, 1 mm EDTA, 1% Triton X-100, 1 mM PMSF and protease inhibitor pellet (Roche)) and twice with wash buffer (100 mM Tris (pH 8), 0.5% Nonidet P-40, 1 mM EDTA, 500 mM NaCl, 250 mM LiCl, 1 mM PMSF and protease inhibitor pellet (Roche)). Real-time qPCR reactions were carried on using SYBR green method. Results shown as fold enrichment at three native subtelomeres (Tel1L, Tel6R and Tel15L) compared to a control (ctrl) late replicating region on Chromosome V (469104–469177) [49, 50]. Primer sequences are listed in S2 Table.

### Microscopy

For Rap1-GFP foci imaging, cell were grown to the 5x10^6^ cells/ml at 34°C for 2 hours in synthetic complete (SC) media. Images were captured immediately in 21 Z-stacks of 0.2 μm using Zeiss Axiovert 200 microscope. GFP foci per nucleus were manually counted as a representation for telomere foci. For Sir4 immunofluorescence, cell cultures were grown to the 5x10^6^ cells/ml at 34°C for 2 hours in synthetic complete (SC) media. Cells were immediately fixed using 3.7% formaldehyde and spheroplasted in SK (0.1M KPO_4_/1.2M sorbitol) buffer containing 0.4 mg/ml Zymolase (US, Biological). Spheroplasted cells were fixed on poly-lysine coated coverslips as described previously [84]. Coverslips were blocked in 1% BSA in PBS for 1 hour, then incubated with primary (αMyc, ab9106-100) followed by secondary (Alexa 488; Molecular Probes, Invitrogen) antibodies each for 30 minutes. Coverslips were mounted on microscope slides using vectashield-containing DAPI (Molecular Probes, Invitrogen). Images were taken in 21 Z-stacks of 0.2 μm using Zeiss Axiovert 200 microscope and Z-stack images were flattened and presented in the figures. ImageJ (NIH, USA) was used for adjusting background in both live and immunofluorescence imaging methods.

### Co-immunoprecipitation assay

Strains carrying HA-tagged Nse6 and Myc-tagged Smc5 were grown to the log phase at room temperature and then incubated for 2 hours at 34°C in YPAD media. Cells were lysed with zirconia beads in lysis buffer (50 mm HEPES, 140 mm NaCl, 1 mm EDTA, 1% Triton X-100, 1 mM PMSF and protease inhibitor pellet (Roche)). Cell lysates were incubated with αMyc antibody-coupled Dynabeads (Invitrogen) for 2 hours at 4°C. Immunoprecipitates were washed once with lysis buffer and twice with wash buffer (100 mM Tris (pH 8), 0.5% Nonidet P-40, 1 mM EDTA, and 400 mM NaCl, 1 mM PMSF and protease inhibitor pellet (Roche)), each for 5 minutes. Beads were resuspended in SDS loading buffer and subjected to SDS gel electrophoresis followed by western blotting by αHA (Santa Cruz, F7) and αMyc (9E10) antibodies. The same procedure was performed for Sir4-Nse3 except that lysates were clarified with one round of centrifugation at 13200 rpm before incubating with Myc antibody-coupled beads and immunoprecipitates were washed once with lysis buffer and twice with wash buffer (100 mM Tris (pH 8), 0.5% Nonidet P-40, 1 mM EDTA, and 250 mM LiCl, 1 mM PMSF and protease inhibitor pellet (Roche)). The co-IP between Sir4 and Smc6 was performed in stationary phase cultures without a chromatin spin and with a wash buffer containing 250 mM NaCl rather than 250 mM LiCl.

### Telomere length analysis by Southern blotting

Measurement of telomere length was performed as described in [15]. Cells were grown for 48 hours to stationary phase in liquid YPAD at 34°C and harvested for Southern blotting. Genomic DNA from each strain were digested with *Xho*I and then separated by 1% agarose gel electrophoresis. Denatured DNA was transferred to Amersham Hybond-XL (GE Healthcare Life Sciences) membrane and hybridized with radiolabeled telomeric repeat probe (TG_1-3_/C_1-3_A). Rediprime II DNA Labeling System used to radiolabel telomeric probe (GE).

### Gene expression analysis

Exponentially growing WT and *nse3*-1 cells were incubated for 2 hours at 34°C prior to harvesting by centrifugation and snap freezing in liquid nitrogen. Cells were lysed and mRNA isolation was followed by reverse transcription Complementary DNA (cDNA) was amplified and quantified using the SYBR Green qPCR method. Primers are listed in S2 Table. Fold gene expression represents real time qPCR values relative to WT samples. Gene expression values were normalized to *ACT1* expression as the internal control.

### RNA Extraction and RT-qPCR for TERRA expression analyses

Total RNA was extracted as in [73]. 2 μg of RNA was treated with 4U of DNase I (Thermo-Fisher) for 4 hr at 37°C and then purified by phenol/chloroform extraction. 1μg of DNase-treated RNA was reverse transcribed by using RevertAid Reverse Transcriptase (Thermo Fisher) at 42°C for 1 hr. 0,5 μmol of a C-rich primer (CACCACACCCACACACCACACCCACA) and 0,5 μg of a poly(dT) primer was used for the reverse transcription reaction (RT). 20ng of cDNA was used for the qPCR, which was performed using the qPCR master mix SsoFAST EvaGreen Supermix from Bio-Rad. qPCR was carried out on a Roche LightCycler96. TERRA expression was normalized against *ACT1* mRNA expression using the delta Ct method and than normalized against the WT yeast strain.

## Supporting Information

S1 Table Strains used in this study. (PDF)

S2 Table qPCR primers used in this study. (PDF)

S3 Table Summary of mutant phenotypes. (PDF)

S1 Fig The *nse3*-1 mutants do not synchronize properly, however components of the Smc5/6 complex still interact. (A) Flow cytometry was performed as described in Fig 1. (B) The fold enrichment levels are relative to the late-replicating control region on Chr V for n = 3 experiments with the mean ± SD at the silent mating type locus (*HMR*) and two regions in the rDNA (*NTS1*) and (*NTS2*) [12]. All primers are listed in S2 Table. (C) Co-immunoprecipitation assay was performed by immunoprecipitating Smc5^Myc^ using α-Myc antibody in WT (JC2229), *nse3*-1 (JC2677) and *smc6*-9 (JC2232) cells. Beads were washed in 400mM NaCl, followed by western blotting for Smc5^Myc^ and Nse6^Ha^ components.
(TIFF)

S2 Fig Sir4 Sumoylation in mutant backgrounds. Sumoylated proteins were isolated by Ni-NTA affinity purification of His-Smt3 as described previously [48, 57, 80, 85] followed by western blotting with αMyc antibodies to visualize sumoylated proteins in cells containing Myc-tagged Sir4 with un-tagged Smt3 wild type (JC3433), or His8-tagged Smt3 in wild type (JC3823), *siz2*Δ (JC3822) *nse5*-ts1 (JC3851) and *mms21*-11 (JC3824).
(TIFF)

S3 Fig TPE measurements from the *URA3* reporter at Telomere VII L. TPE was determined in strains with the *URA3* reporter at the *adh4* locus of Chromosome VIIL. Overnight cultures were spotted onto SC (complete medium) and SC + .1% 5-FOA plates and photographed after incubation at 25C and 34C in wild type (JC1991), *sir4*Δ (JC3818), *nse3*-1(JC3860), *nse3*-1 *sir4*Δ (JC3870), rif2Δ (JC3852), *sir4*Δ *rif2*Δ (JC3872), *nse3*-1 *rif2*Δ (JC3861), *nse3*-1 *rif2*Δ s*ir4*Δ (JC3871) isogenic strains.
(TIFF)

S4 Fig Transcription at sub-telomeric genes in *smc6*-9 mutants. Levels of transcription were compared at sub-telomeric genes *CHA1*, *VAC17* and *YR043C* as described in Fig 4 in wild type (JC470), *sir4*Δ (JC3737), *smc6*-9 (JC3039), and *sir4*Δ *smc6*-9 (JC3925). Expression values are mRNA levels relative to *ACT1* and normalization to wild type cells. Error bars represent ± SD of n = 3 experiments.
(TIFF)

S5 Fig ChIP performed on Rap1^Myc^ and Rif1^Myc^ and in non-tagged (nt) strains. ChIP was perform with Chromatin immunoprecipitation (ChIP) was performed on (A) Rap1^Myc^ in wild type (JC2381) and *nse3*-1 (JC3272), (B) Rif1^Myc^ in wild type (JC3277) and *nse3*-1 (JC3295), (C) α Myc in non-tagged wild type (JC470) and *nse3*-1 (JC3607) cells and (D) α FLAG in non-tagged wild type (JC470), *rif1*Δ (JC3448), and *rif2*Δ (JC2992) cells. The mean ± SD of the fold enrichment at three native subtelomeres (Tel1L, Tel6R and Tel15L) are normalized to the negative ctrl region described in Fig 1F. No statistically significant differences were calculated after a two-tailed *t*-test for Rap1^Myc^ ChIP between wild type and *nse3*-1, the p values < .05 = 0.47 (Tel1), 0.28 (Tel6R), and 0.35 (Tel15L), or for Rif1^Myc^.
(TIFF)

S6 Fig Rif1, Rif2 and Smc6 recruitment at native telomeres in various mutant cells. (A) Yeast-two Hybrid analysis was performed as previously described [48]. *NSE3* full-length, *nse3*(1–150)—N-terminal end, *nse3*^(150–300)^—C-terminal end, or the *nse3*-1 mutant were cloned into bait plasmid (pEG202) and *RIF2* into prey plasmid (pJG4-6) [86]. Plasmids containing bait and prey along with pSH18034 (LacZ reporter plasmid) were transformed into JC1280 and grown overnight in selective media containing 2% raffinose. Overnight cultures were then divided and growth continued in either 2% galactose or 2% glucose for 6 hours at 30°C. β-galactosidase activity was then measured in permeabilized cells as previously described [48, 87]. (B) Western blots with a-HA and a-LexA shows the expression levels of Rif2^HA^, Nse3^LexA^ full-length, N (Nse3^(1–150)^, C-terminal Nse3^(150–300)^ and Nse3-1 peptides from Y2H vectors
(TIFF)

S7 Fig The *nse3*-1 allele, but not the *smc6*-9 allele shortens the long telomeres in cells lacking *RIF2*. Telomere length is determined for the indicated strains by performing southern blot analysis using radiolabeled poly GT/CA probe as explained in Fig 1F and in the experimental procedures section for wild type (JC470), *rif2*Δ (JC2992), *smc6*-9 (JC3039), and *smc6*-9 *rif2*Δ (JC-2993).
(TIFF)

S8 Fig TERRA expression levels in *rif2Δ* and *nse3*-1 mutants. (A and B) TERRA expression was determined for Y’ at 28°C and 34°C in wild type (JC470), *nse3*-1 (JC3607), *rif2*Δ (JC2992), *nse3*-1 *rif2*Δ (JC3269), *sir4*Δ (JC3737), *nse3*-1 *sir4*Δ (JC3741), and *sir4*Δ *rif2*Δ (JC3738). Statistical significance with p values < .05 (*) or < .01 (**) are reported from a two-tailed *t*-test. The Y’ primers detect TERRA expressed from these telomeres: 8L / 8R / 12L-YP1 / 12R-YP2 / 13L / 15R. The arms of chromosome XII contains two short telomeric Y’ elements, YP1 is more end-proximal and YP2 is more centromere-proximal [75].
(TIFF)

S9 Fig TERRA expression and telomeres length in smc6-9 mutants. (A and B) TERRA expression was determined by RT-qPCR for Tel1R and Tel6R, X only telomeres, at 28C (A) and 34C (B). Statistical significance with p values < .05 (*) or < .01(**) are reported from a two-tailed *t*-test. (C) Telomere length was determined as in Fig 1F by Southern blot analysis on 1μg XhoI-digested genomic DNA hybridized with a radiolabeled poly (GT/CA) probe in wild type (JC470), *sir4*Δ (JC3737), *smc6*-9 (JC3039), and *smc6*-9 *sir4*Δ (JC3925).
(TIFF)

S10 Fig Comparison of ChIP levels for Smc6 at telomeres in *sir4Δ* and *nse3*-1 mutants and wild type cells. (A) Chromatin immunoprecipitation (ChIP) on Smc6^FLAG^ in wild type (JC1594) and *nse3*-1 (JC2630) at 25°C. (B) ChIP comparison of Smc6^FLAG^ in wild type (JC1594), *sir4*Δ (JC3732), *nse3*-1 (JC2630). The enrichment at three native subtelomeres (Tel1L, Tel6R and Tel15L) normalized to the negative control region as described in Fig 1B. The levels of Smc6 are reduced further in *nse3*-1 mutants than *sir4*Δ mutants.
(TIFF)

S11 Fig ChIP of yKu70 at telomeres in *nse3*-1 mutant and wild type cells. Chromatin immunoprecipitation (ChIP) was performed on yKu70^Myc^ in wild type (JC1352) and *nse3*-1 (JC3392). The enrichment at three native subtelomeres (Tel1L, Tel6R and Tel15L) normalized to the negative control region as described in Fig 1B.
(TIFF)
