# Supplementary material for: Smc5/6 Is a Telomere-Associated Complex that Regulates Sir4 Binding and TPE
Source: PLoS Genet. 2016 Aug 26;12(8):e1006268. doi: 10.1371/journal.pgen.1006268 (PMC5001636; doi:10.1371/journal.pgen.1006268)
Supplement: S1 Table — (PDF) [file pgen.1006268.s001.pdf]

S1 Table. Strains used is this study

Strain	***Genotype***	***Source***
JC470	*MAT***a** *ade2-1 trp1-1 his3-11 his3-15 ura3-1 leu2-3 leu2-112 Rad5+ (W303)*	R. Rothstein
JC471	*MAT*α *ade2-1 trp1-1 his3-11 his3-15 ura3-1 leu2-3 leu2-112 Rad5+ (W303)*	R. Rothstein
JC1077	JC1991 with *smc6*-9::*KanMX6*	this study
JC1080	JC1991with *mms21*-11::*LEU2*	this study
JC1280	*MAT*α *his3 trp1 ura3-52 leu2*::*proLEU2-lexAop6*	(Golemis et al, 1996)
JC1427	W303 *MAT***a** with *rad52*Δ::*TRP1*	this study
JC1352	W303 *MAT***a** with y*Ku70*-13Myc::*HIS3*	this study
JC1358	W303 *MAT*α *smc6*-9::*KanMX6*	this study
JC1594	W303 *MAT***a** with Smc6-6HIS-3FLAG::*KanMX6*	this study
JC1822	W303 *MAT*α with Rap1-GFP::*LEU2*	GA-1429; S. Gasser lab
JC1827	JC1822 with *mms21*-11::*LEU2*	this study
JC1879	W303 *MAT***a** with *mms21*-11::*LEU2*	(Zhao & Blobel, 2005)
JC1981	W303 *MAT* α with *mms21*-11:: *LEU2*	this study
JC1991	W303 MAT**a** with *adh4*::*URA3* at Tel VII-L	GA-1053; S. Gasser Lab
JC2075	JC1594 with *mms21*-11::*LEU2*	this study
JC2229	*MAT* **a**/α Smc5-13Myc::*KanMX6*; Nse6-6HA::*TRP1*, pep4::*LEU2*	(Bustard et al, 2012)
JC2232	JC2229 *smc6*-9::*KanMX6*	(Bustard et al, 2012)
JC2380	W303 *MAT***a** with Rif2-13Myc::*HIS3*	YU348; D. Shore Lab
JC2381	W303 *MAT***a** Rap1-13Myc::*HIS3*	this study
JC2630	JC1594 with *nse3*-1::*URA3*	this study
JC2671	W303 *MAT***a** with Sir4-13Myc::*KanMX6*	GA-1275; S. Gasser Lab
JC2677	JC2229 *with nse3*-1::*URA3*	this study
JC2710	JC1822 with *smc6*-9::*KanMX6*	this study
JC2754	JC1594 with *rif1*Δ::*HIS3*	this study
JC2823	W303 *MAT***a** with Nse3-6HA::*KanMX6*	this study
JC2907	JC3433 with *smc6*-9::*KanMX6*	this study
JC2992	W303 *MAT***a** with *rif2*Δ*::NatRMX4*	this study
JC2993	JC2992 with *smc6*-9::*KanMX6*	this study
JC3032	W303 *MAT*α with *nse3*-1::*HYG*	this study
JC3039	W303 *MAT***a** with *smc6*-9::*KanMX6*	Y11279; C. Boone Lab
JC3041	JC1822 with *nse3*-1::*URA3*	this study
JC3074	JC1594 with *rif2*Δ::*HIS3*	this study
JC3083	JC2671 with *nse3*-1::*URA3*	this study
JC3087	JC2671 with *smc6*-9::*KanMX6*	this study
JC3235	JC2380 with *nse3*-1::*HYG*	this study
JC3269	W303 *MAT***a** with *nse3*-1::*URA3*, *rif2*Δ::*KanMX6*	this study
JC3272	JC2381 with *nse3*-1::*HYG*	this study
JC3277	W303 *MAT***a** with Rif1-13MYC::*HIS3*	this study
JC3295	JC3277 with *nse3*-1::*HYG*	this study
JC3392	JC1352 with *nse3*-1::*HYG*	this study
JC3433	W303 *MAT***a** with Sir4-13Myc::*KanMX6*	this study
JC3448	W303 *MAT*α with *rif1*Δ::*HIS3*	GA-3619; S. Gasser Lab
JC3452	JC3433 with *nse3*-1::*URA3*	this study
JC3597	JC3433 with *mms21*-11::*LEU2*	this study

JC3603	JC1427 with *rif2*Δ:: *NatRMX4*	this study
JC3607	W303 *MAT***a** with *nse3*-1::HYG	this study
JC3623	JC3448 with *nse3*-1::*HYG*	this study
JC3627	JC1427 with *rif2*Δ::*KanMX6*, *nse3*-1::*HYG*	this study
JC3629	JC1427 with *nse3*-1::*HYG*	this study
JC3720	JC3728 with *sir4*Δ::*HIS3*	this study
JC3728	W303 *MAT***a** with Smc5-6HIS-10FLAG::*KanMX4*	this study
JC3732	JC1594 with *sir4*Δ::*HIS3*	this study
JC3736	JC2671 with Nse3-6HA::*KanMX6*	this study
JC3737	W303 *MAT***a** with *sir4*Δ::*HIS3*	this study
JC3738	JC3737 with *rif2*Δ::*NatRMX4*	this study
JC3741	JC3737 with *nse3*-1::*HYG*	this study
JC3818	JC1991 with *sir4*Δ::*TRP1*	this study
JC3822	JC3823 siz2Δ:: *HIS3*	this study
JC3823	W303 *MAT***a** *SMT3::8HIS-Smt3::TRP1;* Sir4-13Myc::*KanMX6*	this study
JC3824	JC2823 with *mms21*-11::*LEU2*	this study
JC3849	JC3433 with *nse3-1*::HYG	this study
JC3851	JC2823 with *nse3*-1::*URA3*	this study
JC3852	JC1991 with *rif2*Δ::*KanMX6*	this study
JC3853	JC2671 with Smc6-6HIS-3FLAG::*KanMX6*	this study
JC3860	JC1991 with *nse3*-1::*HYG*	this study
JC3861	JC1991 with *nse3*-1::*HYG; rif2*Δ::*KanMX6*	this study
JC3870	JC1991 with *nse3*-1::*HYG; sir4*Δ::*HIS3*	this study
JC3871	JC1991 with *nse3*-1::*HYG; sir4*Δ::*HIS3; rif2*Δ::*KanMX6*	this study
JC3872	JC1991 with *sir4*Δ::*HIS3; rif2*Δ::*KanMX6*	this study
JC3925	JC3039 with *sir4*Δ::*HIS3*	this study
