# Supplementary material for: Smc5/6 Is a Telomere-Associated Complex that Regulates Sir4 Binding and TPE
Source: PLoS Genet. 2016 Aug 26;12(8):e1006268. doi: 10.1371/journal.pgen.1006268 (PMC5001636; doi:10.1371/journal.pgen.1006268)
Supplement: S2 Table — (PDF) [file pgen.1006268.s002.pdf]

S2 Table. qPCR primers used in this study

Gene	primer name	sequence	
		**Expression analysis**	
CHA1		CACTCACGATAACCTGGGCA	
		TTTTCGCTAGTTCTGGCGGT	
VAC17		GCAAGGAACAACAACGCAGA	
		ACCATACACAGACGTTGCCT	
YIR043C		AGCTGCGGTGTTTACAAGT	
		ACTACCGGAAACAAGAAACGTG	
ACT1		TGTCCTTGTACTCTTCCGGT	
		CCGGCCAAATCGATTCTCAA	
		**ChIP analysis**	
Tel 01L	C1555	CAGCCCTAATCTAACCCTGG	S. Gasser lab
	C1556	GTATGGTGAGTAGGTCATG	
Tel 06R	C1557	GTGTGTAGTGATCCGAACTCAGT	S. Gasser lab
	C1558	CTAGCATATTGATATGGCGTACGCACACGT	
Tel 15L	C1559	CCCTAATCTAACCCTGTCCAACCTGTCT	S. Gasser lab
	C1560	TGGTATATACTATAGCATCCGTGGGC	
ZN	C1275	GCACTTAATTGGCGTAAGCTG	(Tittel-Elmer et al, 2012)
	C1276	TCGCAGGAGCATATTTCGTA	
HMR	C1606	TTGTTTTTCGGGCTCATTCTTT	This study
	C1607	ACAATAACAATCGCTCCAGAATTAGC	
NTS1	C1602	TGATGATGGCAAGTTCCAGA	(Torres-Rosell et al., 2005)
	C1603	CTTATTCCTTCCCGCTTTCC	
NTS2	C1604	TGCAAAAGACAAATGGATGG	(Torres-Rosell et al., 2005)
	C1605	GCACCTTTTCCTCTGTCCAC	
		**TERRA analysis**	
Telomere AS oligo		CACCACACCCACACACCACACCCACA	P. Chartrand lab
Y'		GGCTTGGAGGAGACGTACATG	P. Chartrand lab
		CTCGCTGTCACTCCTTACCCG	
Tel 06R		GTCATGGGGCGCAATGGAGTG	P. Chartrand lab
		TAGCATATTGATATGGCGTACGC	
Tel 01L		CGGTGGGTGAGTGGTAGTAAGTAGA	P. Chartrand lab
		CATCCTAACACTACCCTAACACAG	
Act1		TTCCAGCCTTCTACGTTTCC	P. Chartrand lab
		ACGACGTGAGTAACACCATC	
