# Supplementary material for: Smc5/6 Is a Telomere-Associated Complex that Regulates Sir4 Binding and TPE
Source: PLoS Genet. 2016 Aug 26;12(8):e1006268. doi: 10.1371/journal.pgen.1006268 (PMC5001636; doi:10.1371/journal.pgen.1006268)
Supplement: S3 Table — (PDF) [file pgen.1006268.s003.pdf]

S3 Table. Summary of mutant phenotypes compared to wild type

	Clustering intact	Smc5/6 at Telomeres	Sir4 at Telomeres	Silencing- TPE *(URA3* reporter at TELVII)	Telomere length
wild type	(Gotta et al., 1996) (Klein et al., 1992) (Palladino et al., 1993)	Torres-Rosell et al., 2005)	(Strahl-Bolsinger et al., 1997)	+	
smc6-9	like WT	n.d.	like WT	+	like WT
nse3-1	-	↓↓	↓	-	↓
mms21-11	- (Zhao & Blobel, 2005)	like WT	↓	+ (Zhao & Blobel, 2005)	↑ (Zhao & Blobel, 2005)
*sir4*Δ	- (Palladino et al., 1993)	↓	none	- (Aparicio *et al.*, 1991)	↓

Aparicio OM, Billington BL, Gottschling DE (1991) Modifiers of position effect are shared between telomeric and silent mating-type loci in S. cerevisiae. *Cell* **66:** 1279-1287

Gotta M, Laroche T, Formenton A, Maillet L, Scherthan H, Gasser SM (1996) The clustering of telomeres and colocalization with Rap1, Sir3, and Sir4 proteins in wild-type Saccharomyces cerevisiae. *J Cell Biol* **134:** 1349-1363

Klein F, Laroche T, Cardenas ME, Hofmann JF, Schweizer D, Gasser SM (1992) Localization of RAP1 and topoisomerase II in nuclei and meiotic chromosomes of yeast. *J Cell Biol* **117:** 935-948

Palladino F, Laroche T, Gilson E, Axelrod A, Pillus L, Gasser SM (1993) SIR3 and SIR4 proteins are required for the positioning and integrity of yeast telomeres. *Cell* **75:** 543-555

Strahl-Bolsinger S, Hecht A, Luo K, Grunstein M (1997) SIR2 and SIR4 interactions differ in core and extended telomeric heterochromatin in yeast. *Genes Dev* **11:** 83-93

Zhao X, Blobel G (2005) A SUMO ligase is part of a nuclear multiprotein complex that affects DNA repair and chromosomal organization. *Proc Natl Acad Sci U S A* **102:** 4777-4782
