# Supplementary figures and images for: Smc5/6 Is a Telomere-Associated Complex that Regulates Sir4 Binding and TPE
Source: PLoS Genet. 2016 Aug 26;12(8):e1006268. doi: 10.1371/journal.pgen.1006268 (PMC5001636; doi:10.1371/journal.pgen.1006268)

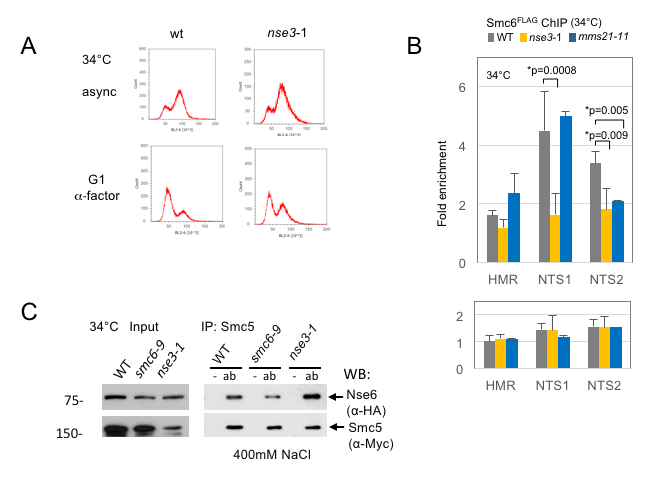

Supplement: S1 Fig — (A) Flow cytometry was performed as described in Fig 1. (B) The fold enrichment levels are relative to the late-replicating control region on Chr V for n = 3 experiments with the mean ± SD at the silent mating type locus (HMR) and two regions in the rDNA (NTS1) and (NTS2) [12]. All primers are listed in S2 Table. (C) Co-immunoprecipitation assay was performed by immunoprecipitating Smc5Myc using α-Myc antibody in WT (JC2229), nse3-1 (JC2677) and smc6-9 (JC2232) cells. Beads were washed in 400mM NaCl, followed by western blotting for Smc5Myc and Nse6Ha components. (TIFF) [file pgen.1006268.s004.tiff]

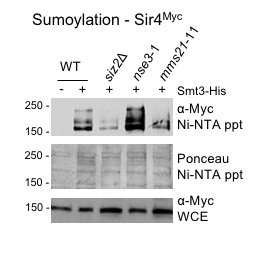

Supplement: S2 Fig — Sumoylated proteins were isolated by Ni-NTA affinity purification of His-Smt3 as described previously [48, 57, 80, 85] followed by western blotting with αMyc antibodies to visualize sumoylated proteins in cells containing Myc-tagged Sir4 with un-tagged Smt3 wild type (JC3433), or His8-tagged Smt3 in wild type (JC3823), siz2Δ (JC3822) nse5-ts1 (JC3851) and mms21-11 (JC3824). (TIFF) [file pgen.1006268.s005.tiff]

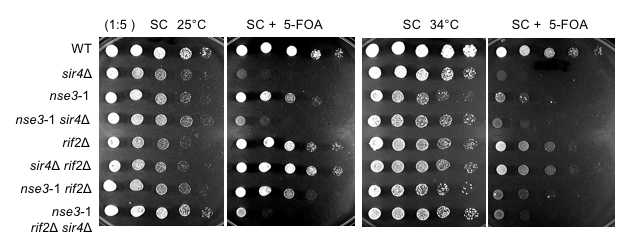

Supplement: S3 Fig — TPE was determined in strains with the URA3 reporter at the adh4 locus of Chromosome VIIL. Overnight cultures were spotted onto SC (complete medium) and SC + .1% 5-FOA plates and photographed after incubation at 25C and 34C in wild type (JC1991), sir4Δ (JC3818), nse3-1(JC3860), nse3-1 sir4Δ (JC3870), rif2Δ (JC3852), sir4Δ rif2Δ (JC3872), nse3-1 rif2Δ (JC3861), nse3-1 rif2Δ sir4Δ (JC3871) isogenic strains. (TIFF) [file pgen.1006268.s006.tiff]

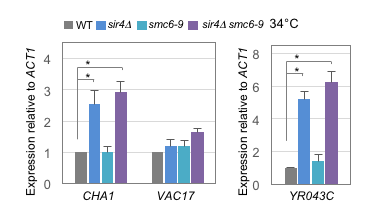

Supplement: S4 Fig — Levels of transcription were compared at sub-telomeric genes CHA1, VAC17 and YR043C as described in Fig 4 in wild type (JC470), sir4Δ (JC3737), smc6-9 (JC3039), and sir4Δ smc6-9 (JC3925). Expression values are mRNA levels relative to ACT1 and normalization to wild type cells. Error bars represent ± SD of n = 3 experiments. (TIFF) [file pgen.1006268.s007.tiff]

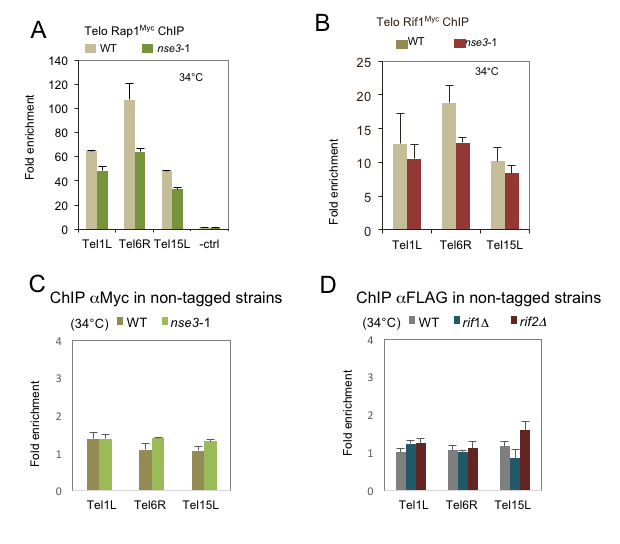

Supplement: S5 Fig — ChIP was perform with Chromatin immunoprecipitation (ChIP) was performed on (A) Rap1Myc in wild type (JC2381) and nse3-1 (JC3272), (B) Rif1Myc in wild type (JC3277) and nse3-1 (JC3295), (C) α Myc in non-tagged wild type (JC470) and nse3-1 (JC3607) cells and (D) α FLAG in non-tagged wild type (JC470), rif1Δ (JC3448), and rif2Δ (JC2992) cells. The mean ± SD of the fold enrichment at three native subtelomeres (Tel1L, Tel6R and Tel15L) are normalized to the negative ctrl region described in Fig 1F. No statistically significant differences were calculated after a two-tailed t-test for Rap1Myc ChIP between wild type and nse3-1, the p values < .05 = 0.47 (Tel1), 0.28 (Tel6R), and 0.35 (Tel15L), or for Rif1Myc. (TIFF) [file pgen.1006268.s008.tiff]

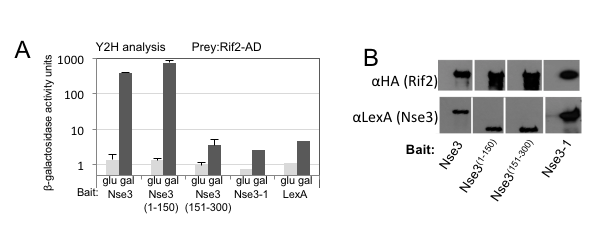

Supplement: S6 Fig — (A) Yeast-two Hybrid analysis was performed as previously described [48]. NSE3 full-length, nse3(1–150)—N-terminal end, nse3(150–300)—C-terminal end, or the nse3-1 mutant were cloned into bait plasmid (pEG202) and RIF2 into prey plasmid (pJG4-6) [86]. Plasmids containing bait and prey along with pSH18034 (LacZ reporter plasmid) were transformed into JC1280 and grown overnight in selective media containing 2% raffinose. Overnight cultures were then divided and growth continued in either 2% galactose or 2% glucose for 6 hours at 30°C. β-galactosidase activity was then measured in permeabilized cells as previously described [48, 87]. (B) Western blots with a-HA and a-LexA shows the expression levels of Rif2HA, Nse3LexA full-length, N (Nse3(1–150), C-terminal Nse3(150–300) and Nse3-1 peptides from Y2H vectors (TIFF) [file pgen.1006268.s009.tiff]

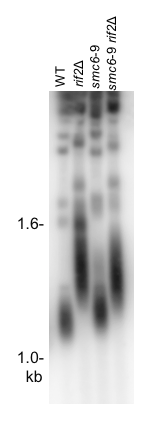

Supplement: S7 Fig — Telomere length is determined for the indicated strains by performing southern blot analysis using radiolabeled poly GT/CA probe as explained in Fig 1F and in the experimental procedures section for wild type (JC470), rif2Δ (JC2992), smc6-9 (JC3039), and smc6-9 rif2Δ (JC-2993). (TIFF) [file pgen.1006268.s010.tiff]

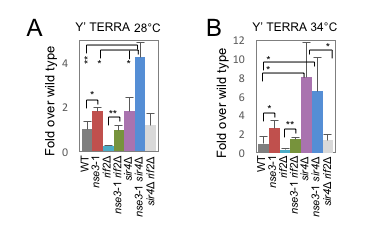

Supplement: S8 Fig — (A and B) TERRA expression was determined for Y’ at 28°C and 34°C in wild type (JC470), nse3-1 (JC3607), rif2Δ (JC2992), nse3-1 rif2Δ (JC3269), sir4Δ (JC3737), nse3-1 sir4Δ (JC3741), and sir4Δ rif2Δ (JC3738). Statistical significance with p values < .05 (*) or < .01 (**) are reported from a two-tailed t-test. The Y’ primers detect TERRA expressed from these telomeres: 8L / 8R / 12L-YP1 / 12R-YP2 / 13L / 15R. The arms of chromosome XII contains two short telomeric Y’ elements, YP1 is more end-proximal and YP2 is more centromere-proximal [75]. (TIFF) [file pgen.1006268.s011.tiff]

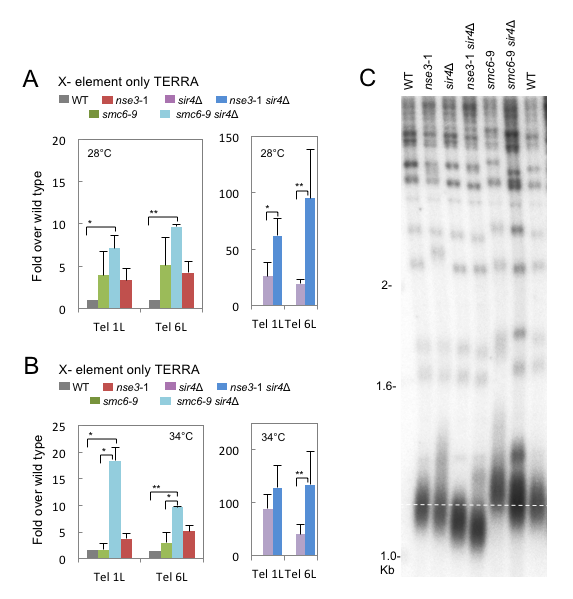

Supplement: S9 Fig — (A and B) TERRA expression was determined by RT-qPCR for Tel1R and Tel6R, X only telomeres, at 28C (A) and 34C (B). Statistical significance with p values < .05 (*) or < .01(**) are reported from a two-tailed t-test. (C) Telomere length was determined as in Fig 1F by Southern blot analysis on 1μg XhoI-digested genomic DNA hybridized with a radiolabeled poly (GT/CA) probe in wild type (JC470), sir4Δ (JC3737), smc6-9 (JC3039), and smc6-9 sir4Δ (JC3925). (TIFF) [file pgen.1006268.s012.tiff]

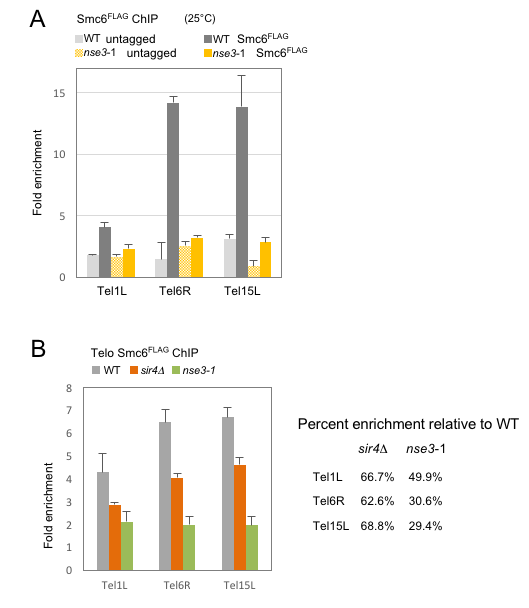

Supplement: S10 Fig — (A) Chromatin immunoprecipitation (ChIP) on Smc6FLAG in wild type (JC1594) and nse3-1 (JC2630) at 25°C. (B) ChIP comparison of Smc6FLAG in wild type (JC1594), sir4Δ (JC3732), nse3-1 (JC2630). The enrichment at three native subtelomeres (Tel1L, Tel6R and Tel15L) normalized to the negative control region as described in Fig 1B. The levels of Smc6 are reduced further in nse3-1 mutants than sir4Δ mutants. (TIFF) [file pgen.1006268.s013.tiff]

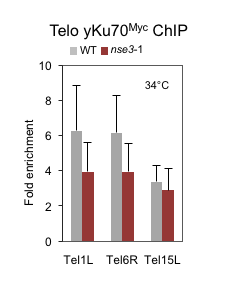

Supplement: S11 Fig — Chromatin immunoprecipitation (ChIP) was performed on yKu70Myc in wild type (JC1352) and nse3-1 (JC3392). The enrichment at three native subtelomeres (Tel1L, Tel6R and Tel15L) normalized to the negative control region as described in Fig 1B. (TIFF) [file pgen.1006268.s014.tiff]
